# An explainable deep learning model for diabetic foot ulcer classification using swin transformer and efficient multi-scale attention-driven network

**DOI:** 10.1038/s41598-025-87519-1

**Published:** 2025-02-03

**Authors:** R. Karthik, Armaano Ajay, Anshika Jhalani, Kruthik Ballari, Suganthi K

**Affiliations:** 1 Centre for Cyber Physical Systems, Vellore Institute of Technology, Chennai, India; 2 School of Computer Science and Engineering, Vellore Institute of Technology, Chennai, India; 3 School of Electronics and Engineering, Vellore Institute of Technology, Chennai, India

**Keywords:** Swin transformer, Diabetic foot ulcer, CNN, Deep learning, Shuffle attention, Biomedical engineering, Health care

## Abstract

Diabetic Foot Ulcer (DFU) is a severe complication of diabetes mellitus, resulting in significant health and socio-economic challenges for the diagnosed individual. Severe cases of DFU can lead to lower limb amputation in diabetic patients, making their diagnosis a complex and costly process that poses challenges for medical professionals. Manual identification of DFU is particularly difficult due to their diverse visual characteristics, leading to multiple cases going undiagnosed. To address this challenge, Deep Learning (DL) methods offer an efficient and automated approach to facilitate timely treatment and improve patient outcomes. This research proposes a novel feature fusion-based model that incorporates two parallel tracks for efficient feature extraction. The first track utilizes the Swin transformer, which captures long-range dependencies by employing shifted windows and self-attention mechanisms. The second track involves the Efficient Multi-Scale Attention-Driven Network (EMADN), which leverages Light-weight Multi-scale Deformable Shuffle (LMDS) and Global Dilated Attention (GDA) blocks to extract local features efficiently. These blocks dynamically adjust kernel sizes and leverage attention modules, enabling effective feature extraction. To the best of our knowledge, this is the first work reporting the findings of a dual track architecture for DFU classification, leveraging Swin transformer and EMADN networks. The obtained feature maps from both the networks are concatenated and subjected to shuffle attention for feature refinement at a reduced computational cost. The proposed work also incorporates Grad-CAM-based Explainable Artificial Intelligence (XAI) to visualize and interpret the decision making of the network. The proposed model demonstrated better performance on the DFUC-2021 dataset, surpassing existing works and pre-trained CNN architectures with an accuracy of 78.79% and a macro F1-score of 80%.

## Introduction

DFU represent a significant and severe complication of diabetes, affecting millions of individuals globally^1^. These ulcers manifest as open sores or wounds predominantly on the feet, resulting from prolonged hyperglycemia that causes nerve damage and peripheral artery disease. The primary symptoms of DFU include persistent pain, swelling, redness and discharge from the affected area. In advanced stages, these ulcers can lead to serious infections such as osteomyelitis, bone infection and potentially necessitate amputation. Approximately 34% of individuals with diabetes will experience a DFU at some point in their lives, indicating that one out of every three people with diabetes will develop this condition^2^. Amputation presents a serious risk for DFU patients, as studies have revealed that 50% of those who undergo amputation die within five years^3^. The challenges posed by DFU extends beyond physical symptoms, significantly impacting healthcare systems and patients’ quality of life. Patients with DFU often endure prolonged hospital stays and frequent outpatient visits. The treatment procedures for DFU often involve a combination of debridement, antibiotic therapy and surgical interventions that can further worsen the already difficult economic conditions of the patients^4^. DFU elevate the risk of other diabetes-related complications including cardiovascular diseases and renal failure, thereby further complicating patient management^5^. Accurate diagnosis and timely intervention are crucial for managing DFU, yet traditional methods present significant challenges.

Manual inspection of ulcers and clinical assessments are time-consuming and require specialized expertise that are prone to subjective errors. Therefore, there is a need for automatic diagnosis systems for DFU detection. Automated techniques can provide consistent and rapid assessments, reducing the burden on healthcare professionals and improving patient outcomes. In recent years, the emergence of Machine Learning (ML) algorithms has facilitated remarkable advancements in the medical field. Instead of relying on predefined instructions, ML can build mathematical models from sample data, allowing computer systems to make predictions by studying algorithms and statistical models^6^. The development of classification models can help physicians diagnose patients’ diseases and related indicators promptly, enabling timely interventions. As a result, these models can contribute to the efficient utilization of medical resources and alleviate the burden on both patients and society.

While ML-based diagnostic methods have shown significant effectiveness, they are not without drawbacks. These systems depend on access to well-curated and diverse datasets for training. Without access to high-quality and diverse training data, ML models may struggle to generalize and effectively capture the disease characteristics. This can lead to suboptimal performance, biased classification and unsatisfactory diagnostic accuracy. Additionally, there is a lack of meta-data such as patient ethnicity, age, sex and foot size which can further complicate the analysis^7^. Data sharing has the potential to facilitate faster data collection, but ethical and privacy issues can hinder its implementation^8^. DL emerges as a potential solution to solve the existing problems of traditional ML methods^9^. Unlike traditional ML methods that rely heavily on manual feature extraction, DL techniques can automatically learn relevant features from large datasets, enhancing accuracy and robustness^10^. DL enables machines to autonomously improve and understand complex patterns, reducing the need for extensive human guidance during model training^11^. The application of DL in DFU classification not only streamlines the diagnostic process but also offers the potential for continuous monitoring and real-time analysis. This can significantly enhance early detection and timely intervention, crucial for preventing severe complications and improving patient prognosis.

Early detection of DFU is therefore crucial, as it can prevent the risks of further complications and ensure timely treatment. This research aims to develop a DL-based classification system for the detection of diabetic foot ulcers. By leveraging DL frameworks, the system seeks to enhance the diagnostic capabilities and enable timely intervention. The goal is to empower healthcare professionals with efficient tools to identify DFU, leading to improved patient care and reduced healthcare costs. The following are the key contributions of this research.

- The proposed dual-track network integrates a transformer-based track for global feature extraction and a custom CNN track for local feature extraction, enabling comprehensive pattern recognition for DFU images. The proposed custom CNN track leverages novel convolution and attention blocks to enhance local feature extraction, leading to improved DFU classification.
- The Global Dilated Attention (GDA) introduced in the proposed work incorporates a global context mechanism to effectively capture long-range dependencies, while a coordinate attention layer is employed to focus on local features by embedding positional information into channel attention. This dual mechanism ensures a balanced extraction of both global and local features, enhancing the performance of the proposed network.
- Shuffle Attention is incorporated to focus on informative regions while reducing computational overhead, enhancing the model’s ability to capture critical features and improve DFU classification accuracy. Grad-CAM is utilized to visualize the model’s decision-making process, offering insights into the key regions that influence DFU classification.

## Related works

Deep learning-based approaches have emerged as a promising solution for the classification and assessment of DFU using medical images. This section presents a comprehensive overview of the different DL techniques that have been leveraged for the classification of DFU. Convolutional Neural Networks (CNNs) have been extensively utilized for classification tasks due to their ability to extract and learn relevant features from image data. CNNs are a type of DL architecture particularly well-suited for processing and analyzing visual information, such as medical images. Transfer learning has also been a valuable technique employed in the context of medical image classification using DL. Transfer learning involves the use of pre-trained models such as ResNet, VGG, Inception, MobileNet, and DenseNet. These models are pretrained on large-scale datasets like ImageNet and serve as a starting point for training a model on a specific task. This approach leverages the knowledge acquired by the pre-trained model which can significantly accelerate the learning process and improve the overall performance, especially when dealing with limited training data^12^.

Ahsan et al. leveraged transfer learning to compare the performance of AlexNet, VGG, GoogLeNet, ResNet, MobileNet, SqueezeNet and DenseNet for infection and ischemia classification^13^. Affine transform techniques were used for the augmentation of input data. ResNet outperformed all other models during testing. Nakka et al. assessed the performance of pre-trained networks for DFU detection using the DFUC-2021 dataset^14^. The study examined five deep neural networks: VGG16, VGG19, ResNet50, ResNet101, and EfficientNetB0. Among the evaluated models, VGG19 achieved better performance compared to the other four networks. Biswas et al. designed the DFU_MultiNet model for DFU classification using three pre-trained CNN models: VGG19, DenseNet201 and NasNetMobile^15^. The three models were trained as three parallel tracks and their outputs were concatenated before being passed on to the classification layers. The proposed framework was developed to offer an efficient DFU classification model that determines the distinction between healthy and ulcerated skin.

In a comparative study, Wu et al. evaluated the performance of two pre-trained models, DenseNet and EfficientNet, for DFU classification^16^. They adopted the Fixmatch technique to leverage the unlabelled data and combined the two models. Additionally, the sharpness-aware minimization optimization algorithm was employed to improve the overall performance of the models. It was observed that the DenseNet model outperformed the EfficientNet model on the test set. Alavi et al. developed a multi-stage model for DFU detection using the DFUC-2021 dataset^17^. In the initial stage, a pre-trained Xception model is chosen as the backbone model for feature extraction. Following that, deep subspace-based descriptors is employed on the Grassmann Manifold to map the image sets into points. To generate representative features from the unlabelled data, Geodesic-Based Relational Representation is utilized through K-medians clustering. Finally, the multi-label random forest algorithm was employed to classify the resulting feature vectors in the last stage. Ahmed et al. proposed an ensemble model that combined multiple models, including EfficientNet B0-B6, ResNet-50, and ResNet-101, for DFU classification^18^. A SoftMax-based custom activation function was proposed to address the class imbalance issue present in the dataset. The ensemble model exhibited robust performance during testing, highlighting its effectiveness in accurately classifying DFU. Bloch et al. presented an ensemble model specifically designed for DFU detection using EfficientNets^19^. Their approach aimed to enhance performance by leveraging pseudo-labeling techniques for unlabeled images and utilizing pix2pixHD Generative Adversarial Network for class-balancing.

Wu et al. proposed an ensemble model for DFU classification that combined three different neural architectures: EfficientNet-B3, DenseNet-201, and BiT-M-R101^20^. Instead of the traditional plurality voting method, a novel voting strategy called voting with expertise was used. This approach allows the ensemble model to consider cases with fewer votes but high probabilities, leading to improved classification accuracy for DFU. The ensemble model demonstrated enhanced performance compared to individual models. Qayyum et al. designed a DFU classification model that utilized two pre-trained vision transformers operating in parallel^21^. The output feature maps from the two transformers were pairwise concatenated. These concatenated outputs then underwent another concatenation step before being passed to the final classification layer. The model demonstrated performance, particularly when average weighted sampling was employed to address the issue of class imbalance. This approach allowed the ensemble model to accurately identify cases with few votes but high probabilities, leading to improved classification accuracy for DFU.

Galdran et al. conducted an evaluation of several models, including ResNeXt50, EfficientNet-B3 (CNN-based models) and vision transformer and data-efficient image transformers (transformer-based models) for DFU classification^22^. The research highlighted that when combined with the Sharpness-Aware loss minimization algorithm, these models achieved improved performance. Notably, the linear combination of BiT-ResNeXt50 emerged as the top-performing model among the evaluated options. Das et al. developed the DFU-SPNet model using three blocks of parallel convolution layers with multiple kernel sizes^23^. The parallel convolution layers had kernel sizes of 1, 3 and 5 respectively with the number of kernels in increasing order of 32, 64 and 128. The output feature maps from the three convolution layers are then concatenated to obtain the enhanced feature maps.

Existing works in DFU classification predominantly rely on pre-trained models, which may not generalize well to the unique features and complexities of DFU. Additionally, there is a notable lack of attention mechanisms in existing studies, which could otherwise improve the focus on critical regions in the images and enhance model performance. Furthermore, many current approaches lack explainability, making it challenging for clinicians to trust and interpret the results. Therefore, there is a growing need for tailored models specifically designed to address the distinctive characteristics of DFU, integrate attention mechanisms and provide explainable outputs. By developing customized models, researchers can enhance the effectiveness and applicability of DFU classification systems, ultimately leading to more accurate and reliable diagnoses.

### Research gaps and motivation

The proposed study aims to address the following research gaps.

1. Existing research works predominantly use single-path architectures which focuses on global feature extraction resulting in loss of information and sub-optimal classification results. There is a lack of models specifically designed for DFU classification.
2. Existing works employ models that lack methods for capturing both multi-scale and cross-channel information, leading to poor model performance and generalization. Additionally, these approaches fail to incorporate architectures that balance computational efficiency with feature extraction capabilities.
3. Current research lacks effective mechanisms to capture both global and spatial channel-specific features, resulting in sub-optimal DFU classification performance. Moreover, these approaches lack effective strategies for integrating positional information into channel attention which is crucial for capturing spatial specific features.
4. There are limited attention mechanisms that simultaneously captures both spatial and channel dependencies. This reduces the model’s discriminative power, preventing it from selectively identifying the most informative regions in the extracted features.

### Research contributions and novelty

The proposed work presents the following key contributions to address existing research gaps.

1. The proposed research work introduces a dual-path architecture featuring a transformer track for global feature extraction and a custom CNN track for local feature extraction. Integrating features from both paths enhances the model’s capability to capture diverse patterns from the images, leading to improved DFU detection.
2. The Light-weight Multi-scale Deformable Shuffle (LMDS) block in the proposed custom CNN model effectively captures features at multiple scales using dynamic kernel sizes and allows for cross channel information flow at a low computational overhead for improved feature extraction and DFU classification.
3. The Global Dilated Attention (GDA) block introduced in the proposed work includes a global context block to identify global features and a co-ordinate attention layer to focus on local features by embedding positional information into channel attention leading to improved DFU classification.
4. Shuffle Attention (SA) is integrated into the architecture of the proposed network to identify the informative regions from the extracted features using spatial and channel attention mechanism as one efficient operation. This enhances the model’s ability to focus on the informative regions and suppress irrelevant ones, thus increasing its discriminative power.

## Proposed system

**Fig. 1:**
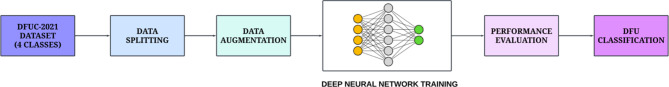
Schematic overview of the proposed approach.

The proposed approach is illustrated in Fig. 1, which outlines the key steps of the methodology, from dataset splitting to performance evaluation. In this research only the labelled images from the DFUC-2021 dataset are considered for model training and evaluation. These labeled samples are then partitioned into three distinct subsets for further processing. To address the class imbalance observed in the dataset, data augmentation techniques are applied to the two classes with fewer images. This approach aims to ensure a more diverse training dataset and enhance model generalization. Subsequently, the proposed model undergoes training followed by performance evaluation.

### Proposed network

**Fig. 2:**
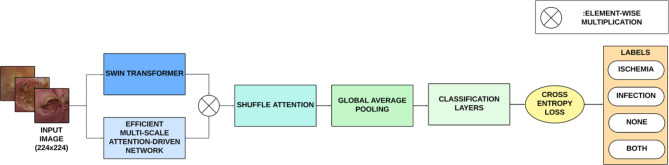
Architectural overview of the proposed network.

The proposed architecture integrates two parallel tracks, the Swin Transformer track and a CNN-based Efficient Multi-Scale Attention-Driven Network (EMADN) track for enhanced feature extraction and learning. This architecture is specifically designed to address the challenges of DFU classification, which requires detecting subtle changes in texture, shape, and regions of interest to enable early diagnosis and treatment. The Swin Transformer track leverages its hierarchical and shifted window-based architecture for improved global feature learning. The transformer track consists of four stages, each containing nested modules of varying input sizes to capture features effectively. This ability to capture global patterns is crucial for recognizing the overall structure and scale of DFUs, which aids in detecting early-stage or widespread ulcers. Conversely, the EMADN track is optimized for capturing low-level features using LMDS and GDA blocks. It employs lightweight and dynamic convolutions for efficient feature extraction and incorporates attention-based modules to refine the captured dependencies. These low-level features are critical for detecting the finer details of DFUs, such as small ulcers or texture variations in skin tissue. In the subsequent stage, the feature maps from both tracks are concatenated using element-wise multiplication. The features from the Swin Transformer track and the features from the EMADN track are multiplied element-wise and then concatenated to form a unified representation of significant features. The resulting fused features are then processed by the Shuffle Attention (SA) module, which integrates channel and spatial attention to identify the most informative regions. Incorporating attention mechanisms into DL architectures has shown improvements in visual intelligence tasks by enhancing the ability of the network to focus on the most relevant features while suppressing irrelevant information^24^. The refined features are then subjected to Global Average Pooling (GAP), which reduces the spatial dimensions and computational requirements. Finally, the feature maps are flattened and passed through a classification layer, with cross-entropy loss. The architecture of the proposed model is depicted in Fig. 2.

#### Swin transformer track

**Fig. 3:**
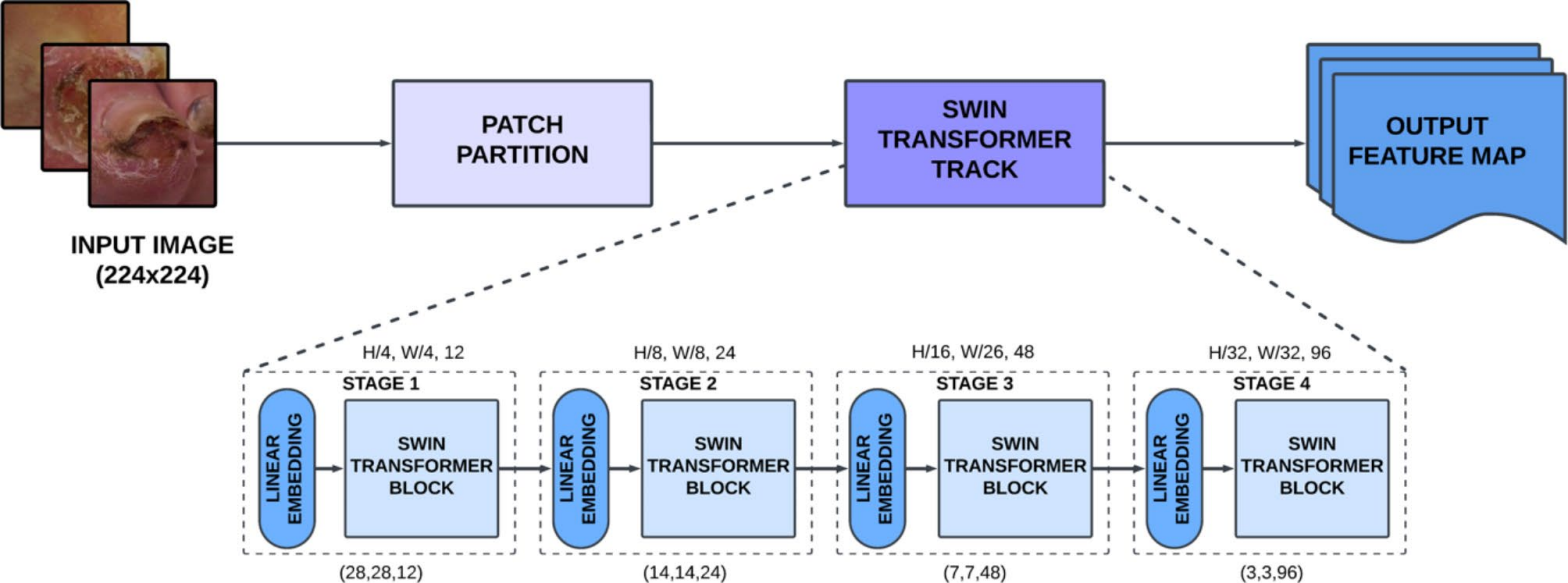
Architecture of the Swin transformer track.

Within this section, we briefly discuss the architecture of the Swin transformer which is employed as the first feature extraction track in the proposed system. Vision transformers are well-known for their ability to capture long-range dependencies from images and therefore a comparative study of five different transformer networks was conducted to identify the best network for DFU classification. A detailed comparison is available in the discussion section. Swin Transformer demonstrated the highest performance and was therefore used as the first feature extraction track for the proposed DFU classification network, improving the identification of global patterns and spatial relationships that are crucial for distinguishing between different types of DFU. The Swin transformer enhances multi-scale feature learning through its hierarchical and shifted window-based architecture. Initially, it constructs a hierarchical representation by dividing the input image into small, non-overlapping patches, which are then merged in successive layers^25^. This process enables the Swin transformer to capture features at different scales more effectively. The four sequential stages of the module generate a feature map with preprocessing modules for image preparation, as illustrated in Fig. 3. Each stage consists of interconnected modules with varying input sizes, contributing to effective feature capture.

The Swin Transformer utilizes a shifted window mechanism to move the window partition between consecutive self-attention layers, enhancing the model’s feature extraction capabilities. The input image is divided into non-overlapping patches through the patch partition module, where each patch is treated is a “token” defined by the concatenation of raw pixel RGB values. A linear embedding layer is integrated to decrease the dimensionality of the patch embeddings, thereby reducing the overall size of each patch while maintaining its spatial dimensions denoted by Height (H) × Width (W). The initial patch merging layer combines features from 2 × 2 neighboring patches, reducing the tokens by a factor of 4. The subsequent layers further process these features, producing output maps of resolution H/8×W/8 for Stage two, H/16×W/16 for Stage three and H/32×W/32 for Stage four. In the Swin transformer, the conventional Multi-Head Self-Attention (MSA) module is replaced with a Shifted Window-based MSA (SW-MSA) module, which incorporates a two-layer Multi-layer Perceptron (MLP) with GELU nonlinearity. Each Swin transformer block includes Layer normalization (LN) before the MLP and MSA, along with residual layers following each component. The computational complexity of each window in the Window-MSA (W- MSA) with a patch size of M × M is given in Eq. (1).

1 $$\:{\varOmega}\:(W - MSA) = 4hwC^{2} + 2M^{2} hwC$$

In the equation given above, $\:\varOmega\:$ is the computational complexity, hw is patch number, M is patch and C is channel features. Cross-window connections optimize computational efficiency across consecutive Swin transformer blocks. The transformer processes patch embeddings through sub-units comprising normalization, attention, and MLP layers. Successive Swin Transformer blocks utilize W-MSA and SW-MSA modules to capture features and spatial relationships in the input data. The output feature maps of successive Swin Transformer blocks, employing shifted window partitioning, can be computed using Eq. (2) to (5).

2 $$\:\hat{z}^{l} = W - MSA\:\left( {LN\:\left( {z^{{l - 1}} } \right)} \right) + z^{{l - 1}} ,$$

3 $$\:{z}^{l}=\:MLP\:\left(LN\:\left({\widehat{z}}^{l}\right)\right)\:+{\widehat{z}}^{l},$$

4 $$\:\hat{z}^{{l + 1}} = \:SW - MSA\:\left( {LN\:\left( {z^{l} } \right)} \right) + z^{l} ,$$

5 $$\:{z}^{l+1}\:=\:MLP\:\left(LN\:\left({\widehat{z}}^{l+1}\right)\right)+{\widehat{z}}^{l+1},\:$$

In the equation given above, $\:{\widehat{z}}^{l}\:$and $\:{z}^{l}$denote the output features of the SW-MSA module and the MLP module, respectively, and l represents the Swin Transformer block. Skip connections are introduced to effectively channel information and capture long-range dependencies. Additionally, a module is employed to down-sample the image, which increases the channel count of the output feature map while reducing the spatial dimensions of the image. Each stage concludes with a patch merging layer, concatenating neighboring patches to down-sample the feature maps. The Swin transformer track outputs 768 extracted features, which are concatenated using element-wise multiplication with the 150 channels extracted by the EMADN track. The fused channels are then passed through advanced modules for improved model performance.

#### Efficient multi-scale attention-driven network track

**Fig. 4:**
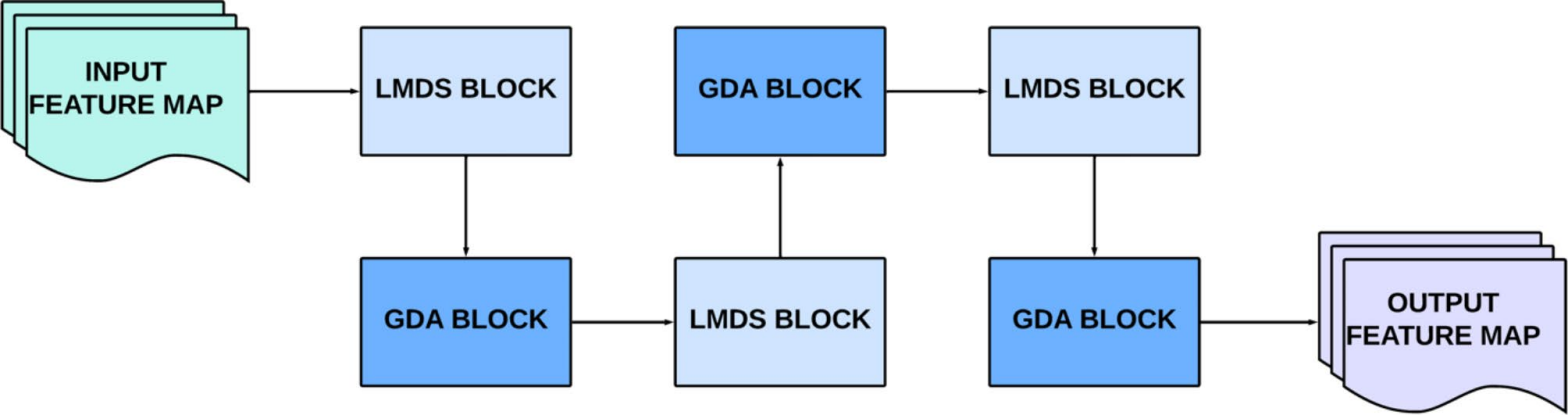
Schematic overview of the EMADN track.

In this section, the architecture of the Efficient Multi-Scale Attention-Driven Network (EMADN) track, which forms the second feature extraction track in the proposed network, is discussed in detail. The EMADN track operates alongside the Swin Transformer track, working synergistically to capture both local and global features from the input DFU images. While the Swin Transformer effectively handles the extraction of high-level contextual features across large regions of the image, the EMADN track is specifically designed to focus on low-level, fine-grained features, which are crucial for distinguishing subtle patterns typical in DFU images, such as variations in skin texture, lesions, or wounds. The specialized blocks within the EMADN track are carefully organized to optimize feature extraction through multi-scale processing and efficient attention mechanisms. These blocks are arranged in a repeated, cascading manner, which helps to amplify the extraction of important features and ensure that the model can capture intricate details. At the end of the EMADN track, the output feature map, consisting of 150 channels, is concatenated with the feature maps from the Swin Transformer track. This combined set of features undergoes further refinement and processing through advanced blocks, enhancing the overall model’s performance in DFU classification. A schematic overview of the EMADN track architecture is shown in Fig. 4.

The EMADN track leverages the sequentially arranged Light-weight Multi-scale Deformable Shuffle (LMDS) and Global Dilated Attention (GDA) blocks for enhanced feature extraction. The LMDS block, is the primary feature extraction block in the EMADN track and leverages the lightweight and efficient Ghost Module (GM) as its first feature extraction layer^26^. Ghost convolution addresses the challenge of limited memory and computational resources, crucial for the deployment of DL models in practical environments, such as DFU diagnosis. The GM reduces computational complexity while improving feature representation. It generates additional feature maps through cheap linear transformations, which unveil hidden information within the intrinsic features. This approach allows the network to extract more informative features while maintaining efficiency, vital for DFU image analysis, where detecting subtle abnormalities is crucial for early diagnosis. Following the GM, the output is passed through two parallel layers with different kernel sizes. The first parallel layer employs a channel shuffle operation, which reorganizes the feature maps into subgroups, encouraging interaction across groups in subsequent layers. This operation promotes a more diverse feature extraction, enhancing the network’s capacity to detect and highlight essential patterns in DFU images that could otherwise be overlooked. This is followed by a Mixed Depth-wise Convolution (MixConv) layer with a kernel size of 3, which enhances feature representation by combining the benefits of multiple kernel sizes in a single convolution operation^27^. The MixConv layer, integral for efficient DFU feature extraction, allows the network to capture both fine and coarse spatial patterns, improving the model’s performance in distinguishing different DFU classes.

**Fig. 5:**
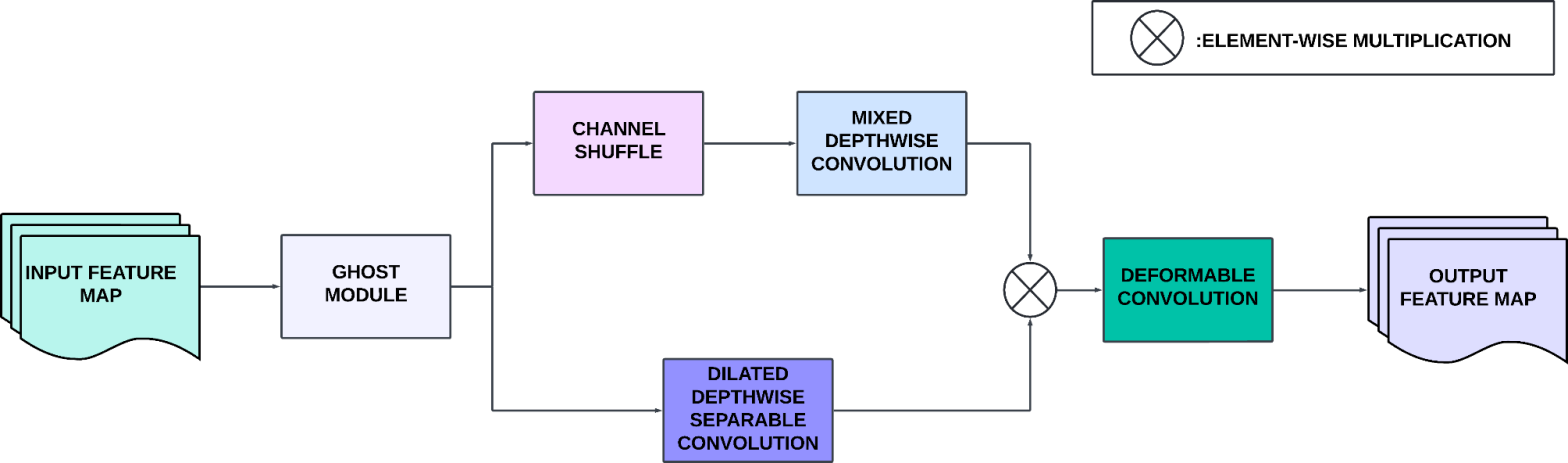
Schematic overview of the LMDS block.

The second parallel layer utilizes a Dilated Depth-wise Convolution (DDSC) with a kernel size of 2, which increases the receptive field while maintaining computational efficiency. The DDSC breaks down the convolution process into individual steps, reducing the computation cost while enhancing feature extraction at multiple spatial scales. The outputs from the MixConv and DDSC layers are then concatenated through element-wise multiplication, ensuring that all the extracted features are combined in a manner that preserves important spatial relationships. Finally, the concatenated feature map is passed through a Deformable Convolution (DC) layer^28^. Deformable convolution introduces 2D offsets to the regular convolution grid, enabling a free-form deformation of the sampling grid. This allows the network to adaptively focus on regions of interest that may be irregular in shape or contain subtle deformities, which is especially relevant when analyzing DFU images where ulcer regions may exhibit varying shapes and sizes. The offsets for the deformable convolution are learned from the preceding feature maps, ensuring that the network focuses on the most informative areas for DFU detection, improving both accuracy and efficiency in classifying different DFU conditions. The DC layer is used instead of a max pooling to prevent information loss during pooling and ensure continuous learning using the improved feature learning capabilities of DC. The architecture of the LMDS block is illustrated in Fig. 5.

The output of the LMDS block is passed to the GDA block for further processing. Attention mechanisms have been extensively explored across various domains, demonstrating exceptional performance in enhancing feature representation and extraction^29^. Drawing inspiration from these advancements, the GDA block was developed, which synergistically integrates the Global Context Block (GCB), Dilated Convolution with Learnable Spacings (DCLS), and Coordinate Attention (CA) mechanisms. This integration allows the network to capture and refine features across both global and spatial dimensions, significantly improving its ability to model complex patterns in DFU images. The GDA block ensures efficient long-range dependency modeling while embedding spatial and positional information, providing a robust framework for diagnosing DFUs. The first layer in the GDA block is the GCB, designed for effective global context modeling^30^. The GCB combines the strengths of Simplified Non-Local (SNL) blocks, which model long-range dependencies, and Squeeze-Excitation (SE) blocks, which improve efficiency with lightweight computations. Within the GCB, global attention pooling is employed using a 1 × 1 convolution and a softmax function to compute attention weights, formulated using Eq. (6).

6 $$\:{\alpha\:}_{j\:}=\frac{{e}^{{W}_{k}{x}_{j}}}{{\sum\:}_{m}{e}^{{W}_{k}{x}_{m}}}$$

Where $\:{W}_{k}$ represents the learnable parameters, and $\:{\alpha\:}_{j\:}$​ denotes the attention weight for the $\:{j}^{th}\:$feature in the feature map. These weights are applied to the feature maps to extract global context features. The extracted context features are then passed through a bottleneck transformation step, defined using Eq. (7).

7 $$\:\delta\:\left(z\right)\:=\:{W}_{{v}_{2}}ReLU\:\left(LN\right({W}_{{v}_{1}}z\left)\right)$$

Where $\:\delta\:\left(z\right)$ represents the transformed context features, $\:{W}_{{v}_{1}}$​ and $\:{W}_{{v}_{2}}$are learnable weight matrices, and LN refers to layer normalization, which improves optimization and regularization. Finally, feature aggregation is performed using a broadcast element-wise addition operation to combine the global context features with the original feature maps. This lightweight design enhances the model’s ability to identify distant, yet relevant, regions, which is essential for detecting complex patterns in DFU images and improving diagnostic accuracy.

Following the GCB, the feature maps are forwarded to the DCLS layer^31^. DCLS extends the dilated convolution concept by introducing learnable spacings between the non-zero elements in the convolution kernel. This method increases the receptive field without significantly increasing the number of parameters, as would happen with larger kernel sizes. The learnable spacings, optimized via backpropagation, enable the model to adaptively focus on relevant regions across the image, a key factor when analyzing DFUs, where lesions may vary widely in size and location across different patient samples. This approach enhances feature extraction while maintaining computational efficiency. Finally, the processed features are passed through the CA mechanism that incorporates positional information into channel attention^32^. Unlike traditional attention mechanisms that often lose spatial details, the CA block preserves positional information by embedding coordinate-based features. The mechanism operates in two stages: coordinate information embedding and coordinate attention generation. In the first stage, pooling operations are applied along the horizontal (H,1) and vertical (1,W) spatial extents to encode long-range dependencies in each direction. For a feature tensor $\:X\in\:\:{R}^{C\times\:H\times\:W}$, the pooled outputs for the $\:{c}^{th}\:$ channel are given using Eq. (8) and Eq. (9).

8 $$\:{z}_{c}^{h}\left(h\right)=\frac{1}{W}\sum\:_{i=1}^{W}{x}_{c}\:(h,i)$$

9 $$\:{z}_{c}^{w}\left(w\right)=\frac{1}{H}\sum\:_{j=1}^{H}{x}_{c}\:(j,w)$$

Where $\:{z}_{c}^{h}\left(h\right)$​ and $\:{z}_{c}^{w}\left(w\right)$​ represent direction-aware feature maps for horizontal and vertical dimensions, respectively. These outputs encode spatial information while maintaining positional awareness. In the second stage, the pooled features are concatenated and passed through a shared 1 × 1 convolutional transformation $\:{F}_{1}$​, resulting in an intermediate feature map as given in Eq. (10).

10 $$\:f=\delta\:\left({F}_{1}\right([{z}^{h},\:{z}^{w}]\left)\right)$$

Where $\:\delta\:$ denotes a non-linear activation function, and $\:[{z}^{h},\:{z}^{w}]$ indicates the concatenation operation. The resulting feature map $\:f$ is split into two tensors, $\:{f}^{h}\in\:\:{R}^{\frac{C}{r}\times\:H}$and $\:{f}^{w}\in\:\:{R}^{\frac{C}{r}\times\:W}$, where *r* is the reduction ratio. These tensors are further transformed using separate 1 × 1 convolutions $\:{F}_{h}\:$and $\:{F}_{w}$​, producing attention weights as illustrated in Eq. (11) and Eq. (12).

11 $$\:{g}_{c}^{h}\left(h\right)\:=\:\sigma\:\left({F}_{h}\right({f}^{h}\left)\right)$$

12 $$\:{g}_{c}^{w}\left(w\right)\:=\:\sigma\:\left({F}_{w}\right({f}^{w}\left)\right)$$

Where $\:\sigma\:$ is the sigmoid activation. These attention weights are expanded along their respective dimensions and applied to the input tensor X to generate the output tensor as given in Eq. (13).

13 $$\:{y}_{c}(i,j)\:=\:{x}_{c}(i,j)\cdot\:{g}_{c}^{h}\left(i\right)\cdot\:{g}_{c}^{w}\left(j\right)$$

**Fig. 6:**
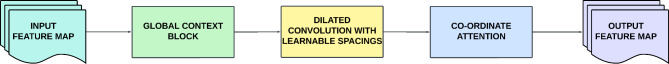
Visual overview of the GDA block.

Therefore, the GDA block efficiently models long-range dependencies while embedding spatial and positional information, enabling the network to focus on key regions in the image. This enhances the identification of critical DFU features, such as ulcer size and shape, essential for accurate diagnosis. Figure 6 provides a visual overview of the GDA block.

#### Shuffle attention (SA)

Channel and spatial attention mechanisms are commonly utilized to improve feature selection by emphasizing the most relevant channels and spatial regions^33^. In the proposed architecture the feature maps from both the tracks are fused using element-wise multiplication and then forwarded to the SA unit for further refinement. The SA unit is a lightweight attention mechanism that integrates both channel and spatial attention into a single block, aiming to enhance the interaction of features^34^. SA operates by dividing the input feature map *X* ∈ *R*^*C×H×W*^ into *G* groups along the channel dimension. Each group, *X*_*k*_ ∈ *R*^*C/G×H×W*^, captures specific semantic responses during training. Within each group, the feature map is divided along the channels into two branches: *X*_*k1*_*and X*_*k2*_, both having dimension *R*^*C/(2G) H×W*^. One branch generates a channel attention map, leveraging the inter-channel relationships to focus on important channels. The channel attention can be mathematically represented using Eq. (14).

14 $$\:{X{\prime\:}}_{k1}=\sigma\:({W}_{1}s+{b}_{1})\cdot\:{X}_{k1}$$

In the equation given above X is feature map, $\:\sigma\:$ is sigmoid activation and s is channel-wise statistics. $\:{W}_{1}\in\:{R}^{C/2G\times\:1\times\:1}$ and $\:{b}_{1}\in\:{R}^{C/2G\times\:1\times\:1}$ are parameters used to scale and shift s. The other branch of SA creates a spatial attention map, exploiting inter-spatial relationships to highlight significant spatial locations. The attention maps, X_C_ and X_S_, have dimensions R^C/G×1 × 1^ and R^H×W×1^, respectively. The final output from spatial attention can be calculated using Eq. (15).

15 $$\:{X{\prime\:}}_{k2}=\sigma\:({W}_{2}.GN({X}_{k2})+{b}_{2})\cdot\:{X}_{k2}$$

In the equation given above *X* is feature map, $\:\sigma\:$ is sigmoid activation and *GN* is group normalization.

$\:{W}_{2}\:$and $\:{b}_{2}$are parameters with shape $\:{R}^{C/2G\times\:1\times\:1}$. After calculating these attention maps, they are combined through element-wise multiplication to form a feature map X’ with dimensions R^C×H×W^. This process enhances each channel’s attention by incorporating its spatial importance. To enable effective information exchange between different sub-features, the channel shuffle operation is applied, which reorders the channels to ensure comprehensive feature interaction.

**Fig. 7:**
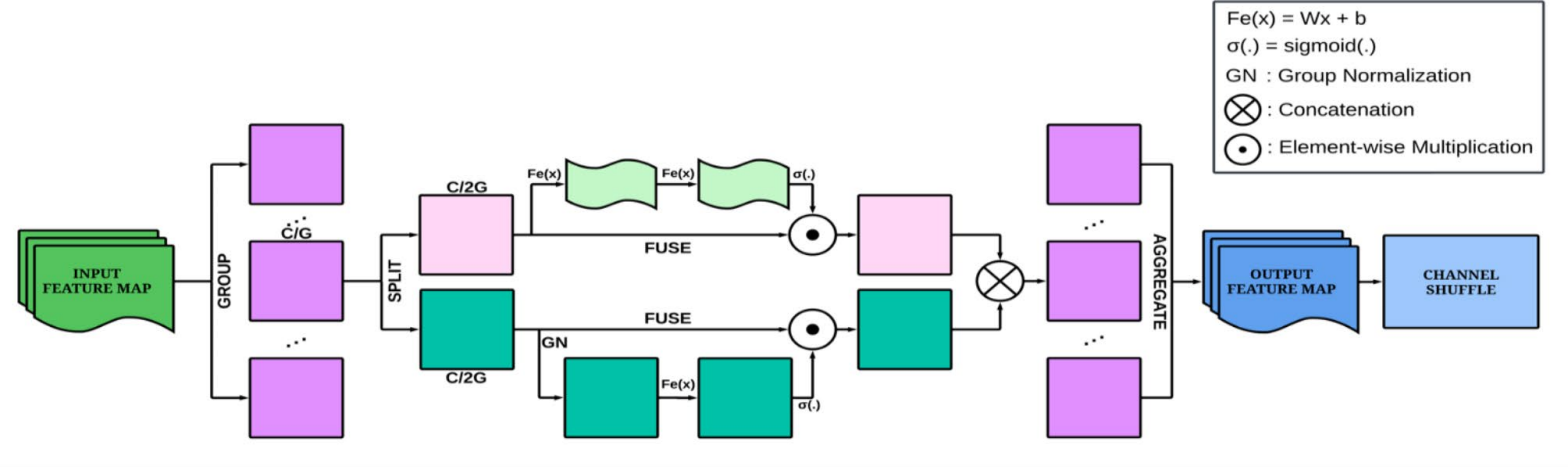
Structural overview of the SA block.

By combining channel and spatial attention with the channel shuffle operation, the SA module significantly improves the model’s ability to process and interpret complex visual information. This is particularly beneficial in the context of DFU classification, where accurate detection of important regions such as the size, shape, and position of ulcers is crucial. The channel attention helps the model focus on the most informative channels, ensuring that critical features are emphasized, while the spatial attention enables the model to highlight relevant spatial regions across the input images. The channel shuffle operation further improves feature interaction, allowing for better integration of multi-scale features, which is essential for handling the varying sizes and complexities of DFUs. Overall, the SA module enhances the model’s performance and reliability in DFU classification by providing a lightweight, efficient way to focus attention on both important features and regions. This leads to improved accuracy, better localization of ulcer regions, and more reliable predictions. A visual overview of the SA module is provided in Fig. 7.

#### Classification

The enhanced feature maps produced by the SA module undergo global average pooling to reduce their spatial dimensions, effectively decreasing the parameters and computational complexity of the network. The pooled features are then flattened and passed through a ReLU-activated classification layer with dropout to prevent overfitting. To train the network, cross-entropy loss is used to optimize the model weights. Cross-entropy loss calculates the divergence between the predicted probability distribution and the actual target distribution. The model is penalized for incorrect predictions, especially those made with high confidence and is rewarded for aligning predictions closely with the true labels. By minimizing the loss, the model iteratively adjusts its parameters to improve its classification performance, thereby enhancing its ability to generalize beyond the training data.

## Results

In this section, we detail the dataset used, the data augmentation methods employed along with the environmental setup for model training. In the subsequent section, the process of hyper-parameter tuning is discussed. Ablation studies is conducted to evaluate the contribution of individual components towards overall model performance. Finally, a comparative performance analysis of the proposed approach is presented.

### Dataset description

**Fig. 8:**
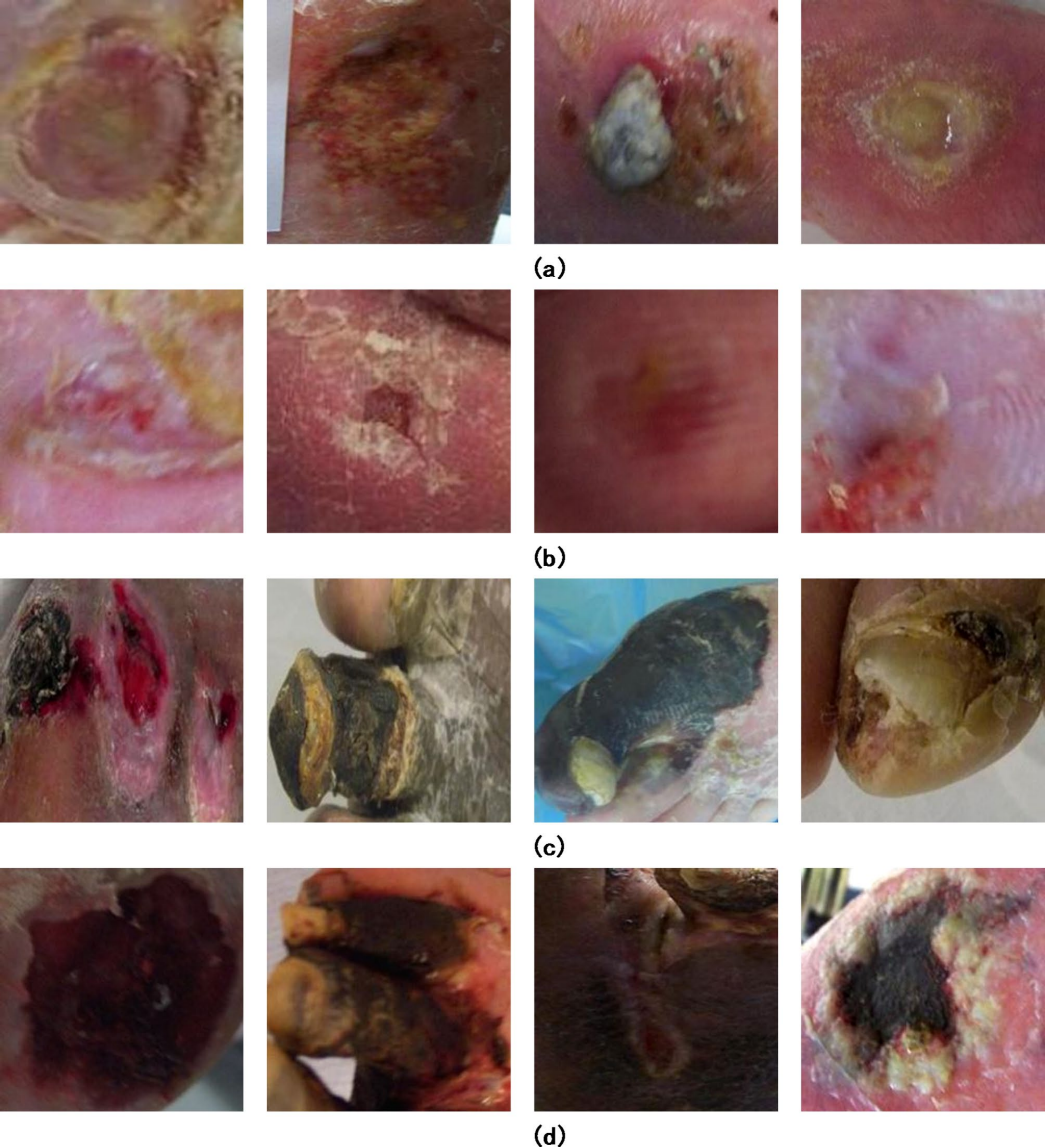
Example images of various disease classes from the DFUC-2021 dataset (**a**) infection (**b**) none (**c**) ischemia (**d**) both.

The DFUC-2021 dataset utilized in this research consists of 5,955 labelled images^35^. For this research, the focus is solely on the labeled DFU images for training and evaluation purposes. These DFU images were obtained from patients at Lancashire Teaching Hospitals using close-up shots of diabetic feet, taken from a distance of around 30–40 cm perpendicular to the ulcer plane. The dataset is organized into four classes: infection, ischemia, none and both. Figure 8 provides visual samples from each class within the DFUC-2021 dataset.

### Data augmentation

This section outlines the various data augmentation methods applied to the dataset for model training. The DFUC-2021 dataset consists of 5,955 labeled images, each of a standard resolution of 224 × 224 pixels. To ensure a balanced and representative dataset for model training, validation and testing the labeled images are divided into three distinct subsets: 60% for training, 20% for validation and 20% for testing. To address the issue of class imbalance and enhance the diversity of the training and validation sets, data augmentation techniques were employed for the ischemia and both classes which had a relatively smaller number of images. Four augmented images were added for every original image of the ischaemia and both classes. This strategic data augmentation process increases the overall dataset size, thereby improving model robustness while reducing the risk of overfitting. The data augmentation methods used include: (1) Randomly flipping the images vertically. (2) Randomly flipping the images horizontally. (3) Randomly rotating the images by an angle of [0°, 90°, 270°]. The class-wise statistics of the dataset is given in Table 1.

**Table 1:** Class-wise statistics for DFUC-2021 dataset.

S. No.	Class name	Before augmentation			After augmentation		
		Train set	Validation set	Test set	Train set	Validation set	Test set
1	None	1631	510	511	1631	510	511
2	Infection	1533	511	511	1533	511	511
3	Ischemia	136	45	46	680	225	46
4	Both	372	124	125	1860	620	125
	Total	3672	1190	1193	5704	1866	1193

### Environment setup

A virtual 16GB Nvidia Tesla P100 GPU provided by Kaggle Cloud was used to conduct all the experiments virtually for the proposed system. Along with the GPU resources, 30 GB of virtual memory was provided by Kaggle, with PyTorch being used for model implementation. Hyper-parameter tuning was carried out during training using Stochastic Gradient Descent (SGD) algorithm. To ensure consistent learning a Step-LR Scheduler was used alongside the SGD algorithm, with cross-entropy employed as the loss function.

### Hyper-parameter tuning

Hyper-parameter tuning is vital for training DL networks, as it significantly improves model generalizability and overall learning capacity. In this research, we focused on optimizing four integral hyper-parameters: (1) dropout rates, (2) learning rate and weight decay, (3) gradient optimizer, and (4) step size for the learning rate scheduler using the grid-search algorithm. We experimented with various values for each hyper-parameter: dropout rates ranging from 0.1 to 0.8, learning rates and weight decay values ranging from 0.1 to 0.00001. ADAM and SGD optimization algorithms were explored and paired with step sizes ranging from 1 to 25 in increments of 5. The results showed that the best model performance was achieved with a dropout rate of 0.6, learning rate of 0.0001 and weight decay set to 0.001. Additionally, the model reached maximum convergence when using SGD with a momentum value of 0.9 was combined with a Step-LR scheduler with a step size of 1 and a gamma decay of 0.99. The detailed hyper-parameter tuning results, obtained using the grid-search algorithm, are provided in Table 2.

**Table 2:** Results of hyperparameter tuning using grid-search.

Hyperparameter	Values	Optimal values
Learning rate	[1, 0.00001]	0.0001
Weight decay	[1, 0.00001]	0.001
Optimiser	[ADAM, SGD]	SGD
Dropout	[0.1, 0.8]	0.6
Step size	[1, 25]	1

### Ablation studies

Ablation experiments were conducted to assess the effectiveness of different components in the proposed architecture. The goal was to identify the most effective combination of these components to enhance model performance. The performance of the (1) Swin Transformer track, (2) EMADN Network track, and (3) Shuffle Attention is evaluated using accuracy, precision, recall and macro F1-score.

#### Analysis of the Swin transformer track

The performance of the Swin transformer track is analysed in this section. Swin transformer leverages shifted windows to capture long-range dependencies efficiently at a reduced computational complexity. The Swin transformer operates through multiple hierarchical stages, progressively refining the representations. This approach improves the ability of the model to capture contextual information and enhances overall performance. Subsequently the output feature map generated by the Swin transformer undergoes adaptive average pooling to reduce dimensionality. The feature maps are then passed through the classification layers, which include a hidden layer for improved performance. The model is then trained for 250 epochs using the SGD optimizer with momentum. The model achieved an accuracy of 68.73% during testing, with an average precision of 67%, recall of 73%, and macro F1-score of 68%. The class-wise metrics are provided in Table 3 for further clarity.

**Table 3:** Class-wise metrics for swin transformer track.

Class	Accuracy (in %)	Precision (in %)	Recall (in %)	F1-Score (in %)
Both	74	74	74	74
Infection	71	64	71	67
Ischemia	52	75	52	62
None	66	74	67	70

#### Analysis of the EMADN track

This section examines the EMADN track’s performance in two configurations: using only the LMDS block and combining it with the GDA block. These experiments assess the individual and combined contributions of local feature extraction and attention modules.

##### Analysis of the EMADN track without GDA block

In the first configuration, only the LMDS block was utilized in the EMADN track for feature extraction. The LMDS block, comprising GM, MixConv, DDSC and DC, efficiently capture multi-scale local features while maintaining computational efficiency. The use of DC and channel shuffling enhanced the network’s ability to focus on regions of interest and promote diverse feature extraction. Despite these strengths, the absence of global context modeling and channel refinement limited the track’s performance, with the model achieving an accuracy of 50.04%, precision of 58%, recall of 44%, and macro F1-score of 37%. Detailed class-wise metrics for this configuration are provided in Table 4.

**Table 4:** Class-wise metrics for EMADN track without GDA block.

Class	Accuracy (in %)	Precision (in %)	Recall (in %)	F1-Score (in %)
Both	8	77	8	14
Infection	12	67	13	22
Ischemia	58	38	59	46
None	96	49	97	65

##### Analysis of the EMADN track with GDA block

When the GDA block was integrated into the EMADN track, it significantly enhanced the feature extraction process by complementing the LMDS block’s local feature learning with global context modeling and refined attention mechanisms. The GDA block, incorporating the GCB, DCLS and CA, captured long-range dependencies, expanded the receptive field, and emphasized critical spatial regions in the feature maps. These advancements led to improved classification performance, with the model achieving an accuracy of 71.17%, precision of 70%, recall of 66%, and macro F1-score of 67%. Detailed class-wise metrics for this configuration are available in Table 5.

**Table 5:** Class-wise metrics for EMADN track with GDA block.

Class	Accuracy (in %)	Precision (in %)	Recall (in %)	F1-Score (in %)
Both	84	69	84	76
Infection	67	71	67	69
Ischemia	39	69	39	50
None	74	72	75	74

#### Analysis of the proposed network without shuffle attention

This section discusses the effectiveness of the proposed network before the SA module was integrated into the network architecture. The proposed system concatenates the output feature maps generated by the Swin transformer track and the DAMFN track for enhanced feature extraction. The network was trained for 250 epochs using the SGD algorithm with momentum and cross-entropy as the loss function. Results show that during testing, the model achieved an accuracy of 78.29%, precision of 79%, recall of 79%, and a macro F1-score of 78%. The class-wise metrics are provided in Table 6 for further analysis. While achieving satisfactory performance, the model underscores the importance of integrating attention-based modules like SA, crucial for capturing and preserving essential spatial relationships.

**Table 6:** Class-wise metrics for dual-track architecture.

Class	Accuracy (in %)	Precision (in %)	Recall (in %)	F1-Score (in %)
Both	84	83	84	83
Infection	74	76	75	76
Ischemia	73	81	74	77
None	79	78	79	78

#### Analysis of the proposed network with shuffle attention

**Fig. 9:**
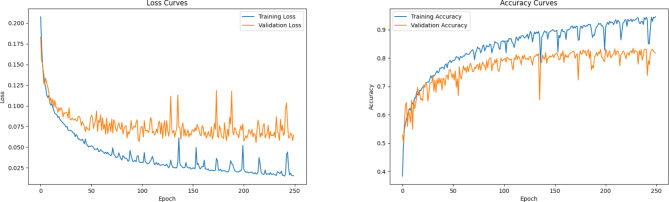
Evaluation of proposed system with SA—loss and accuracy curves.

**Fig. 10:**
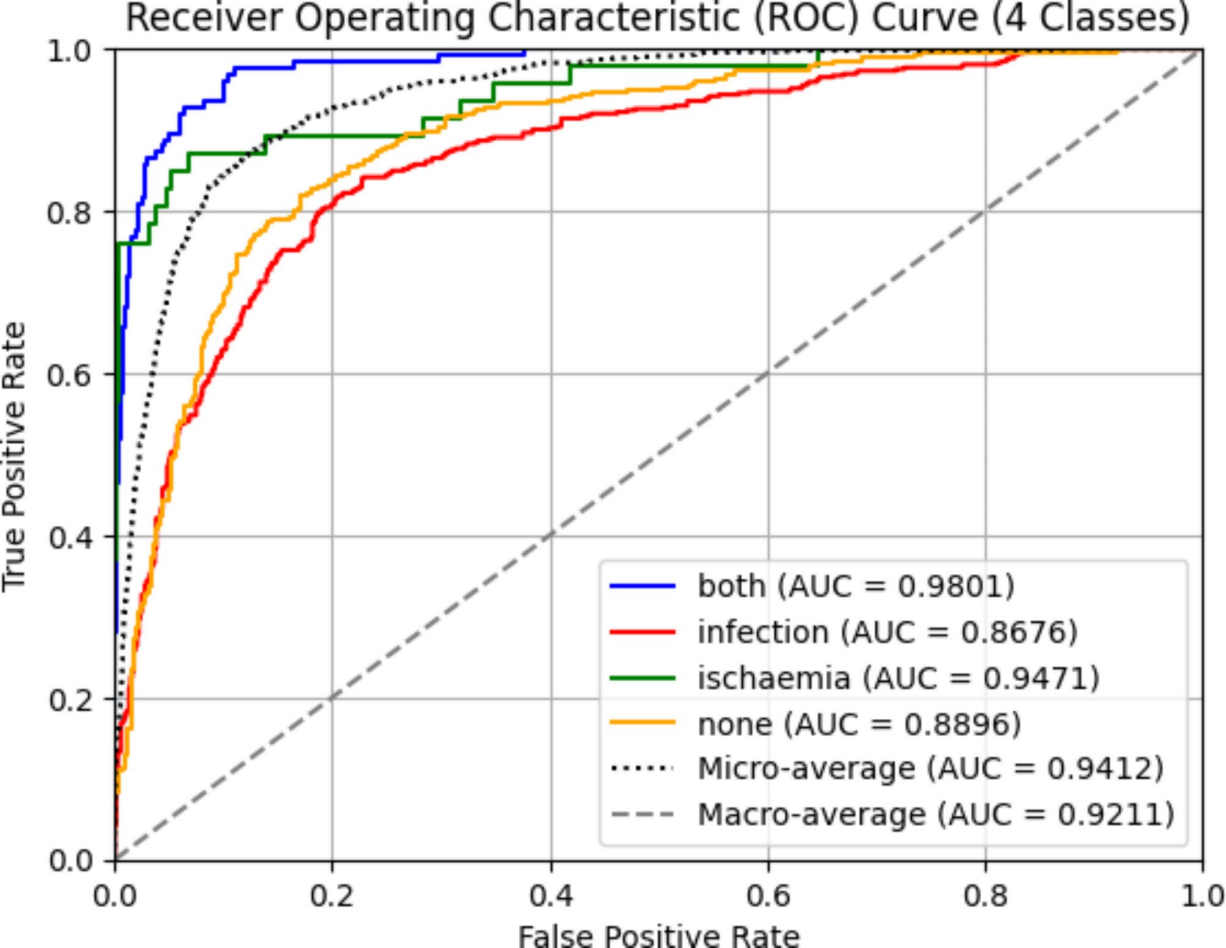
ROC curve for proposed network.

**Fig. 11:**
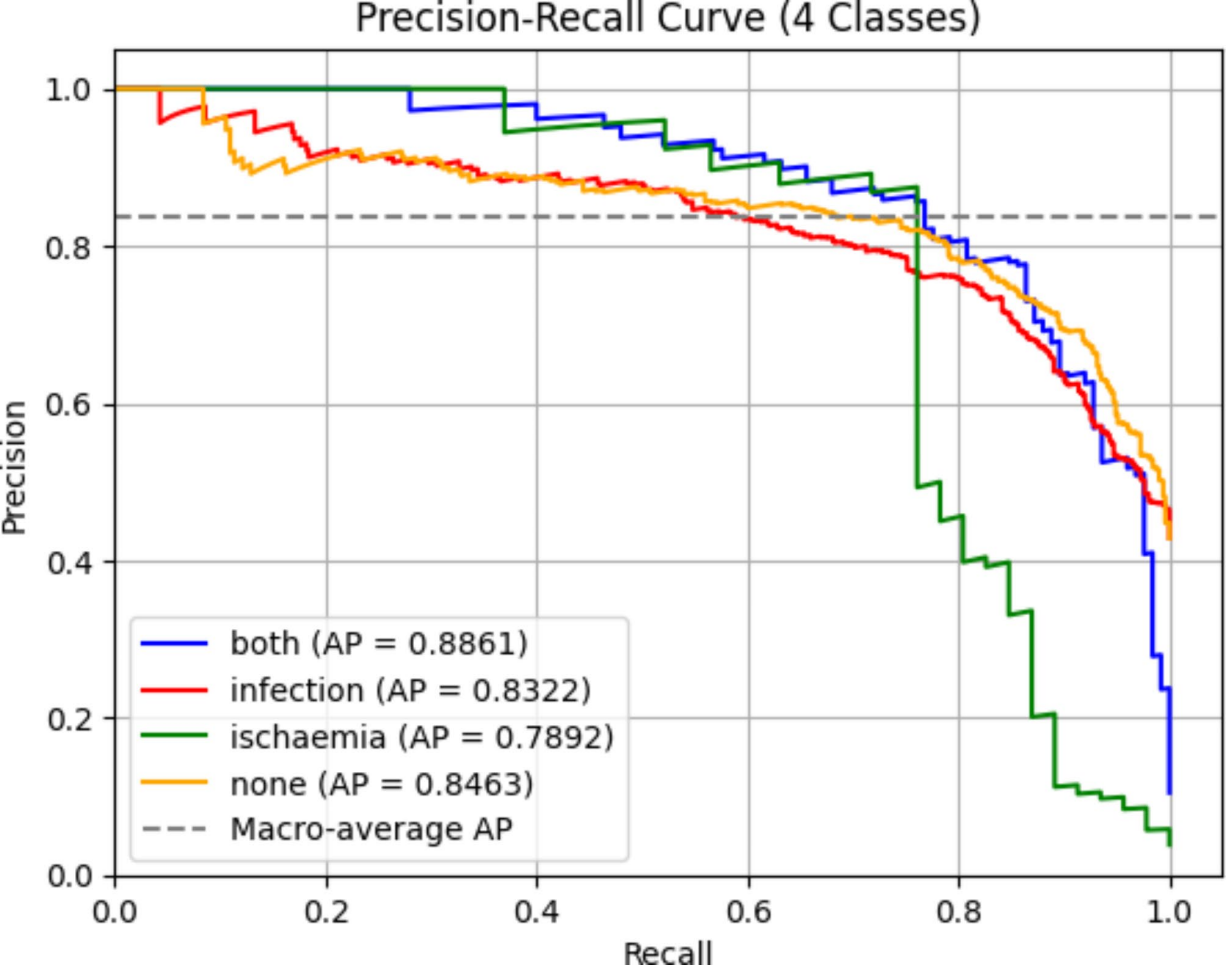
Precision-recall curve for the proposed network.

In this section, we analyze the effectiveness of integrating the SA module into the proposed network. To find the best attention mechanism, we conducted an ablation study of six attention mechanisms. SA not only achieved the best performance but also had the lowest increase in parameters compared to other attention mechanisms, making it a highly efficient choice. The detailed metrics are available in Table 7. SA demonstrates its ability to preserve essential contextual information and relationships within the data, thereby enhancing the model’s performance by capturing intricate patterns. It achieves this with efficient encoding of inter-channel and spatial information, minimizing computational complexity. The network undergoes 250 epochs of training using the SGD optimizer with momentum. The training convergence of the proposed model when SA is integrated is illustrated in Fig. 9. During testing, the model achieves an accuracy of 78.79%. The precision, recall, and F1-score reached 81%, 79%, and 80%, respectively, highlighting the improved performance achieved by the addition of SA. A summary of the conducted ablation studies and their respective metrics is detailed in Table 8. Additionally, class-wise metrics can be found in Table 9. The ROC and precision-recall curves are shown in Figs. 10 and 11, with the macro-average AUC reaching 92.11%.

**Table 7:** Comparison of different attention mechanisms.

Experiments	Number of parameters	Accuracy (in %)	Precision (in %)	Recall (in %)	F1-Score (in %)
Convolutional block attention module	2,92,70,816	72.59	74	75	74
Spatial group-wise enhance	2,91,62,313	76.45	81	74	77
Squeeze and excitation	2,92,70,716	76.53	76	76	76
Efficient channel attention	2,91,65,094	76.70	78	75	76
Triplet attention	2,91,65,289	78.71	78	82	76
Shuffle attention	2,91,61,395	78.79	81	79	80

**Table 8:** Ablation studies summarized.

Experiments	Number of parameters	Training time (in h)	Accuracy (in %)	Precision (in %)	Recall (in %)	F1-Score (in %)
Swin transformer track	27,512,560	6.04	68.73	67	73	68
EMADN track (without GDA)	411,990	5.16	50.04	58	44	37
EMADN track	707,373	3.24	71.17	70	66	67
Proposed network without SA	29,160,477	9.36	78.21	79	79	78
Proposed network with SA	29,161,395	8.38	78.79	81	79	80

**Table 9:** Class-wise metrics for proposed network.

Class	Accuracy (in %)	Precision (in %)	Recall (in %)	F1-Score (in %)
Both	84	83	84	83
Infection	74	76	75	76
Ischemia	75	81	77	79
None	79	81	79	80

## Discussion

A detailed visual analysis of the features generated by the proposed two track network is provided in this section. Additionally, the performance of the proposed system is compared against other state-of-the-art architectures and existing works.

### Grad-CAM visualization

Understanding and interpreting the DL network and its predictions are essential for developing an XAI model. To incorporate this, Gradient-weighted Class Activation Mapping (Grad-CAM) is used to generate heat maps that visualize the salient regions in the DFU images^36^. Grad-CAM leverages the spatial information retained in the convolutional layers of a CNN, which is often lost in fully connected layers. By applying the gradient of the target class’s score with respect to the feature map activations, Grad-CAM highlights the important regions in the input image that contribute to the model’s predictions. The primary advantage of Grad-CAM lies in its ability to provide visual interpretability by identifying key features that influence the classification decision. This feature is particularly critical in medical imaging tasks, as it enables clinicians to cross-verify whether the model’s focus aligns with known pathological patterns. For example, regions associated with infections, ischemia, or both are identified and emphasized. By highlighting important regions that contribute to the model’s predictions, Grad-CAM helps in understanding which parts of the image are most influential in the decision-making process. In medical contexts, such as DFU classification, this interpretability can enhance trust in the model’s outputs by demonstrating alignment with medical reasoning. A visualization of the gradients generated in the last convolutional layer of the proposed network is illustrated in Table 10.

**Table 10:**
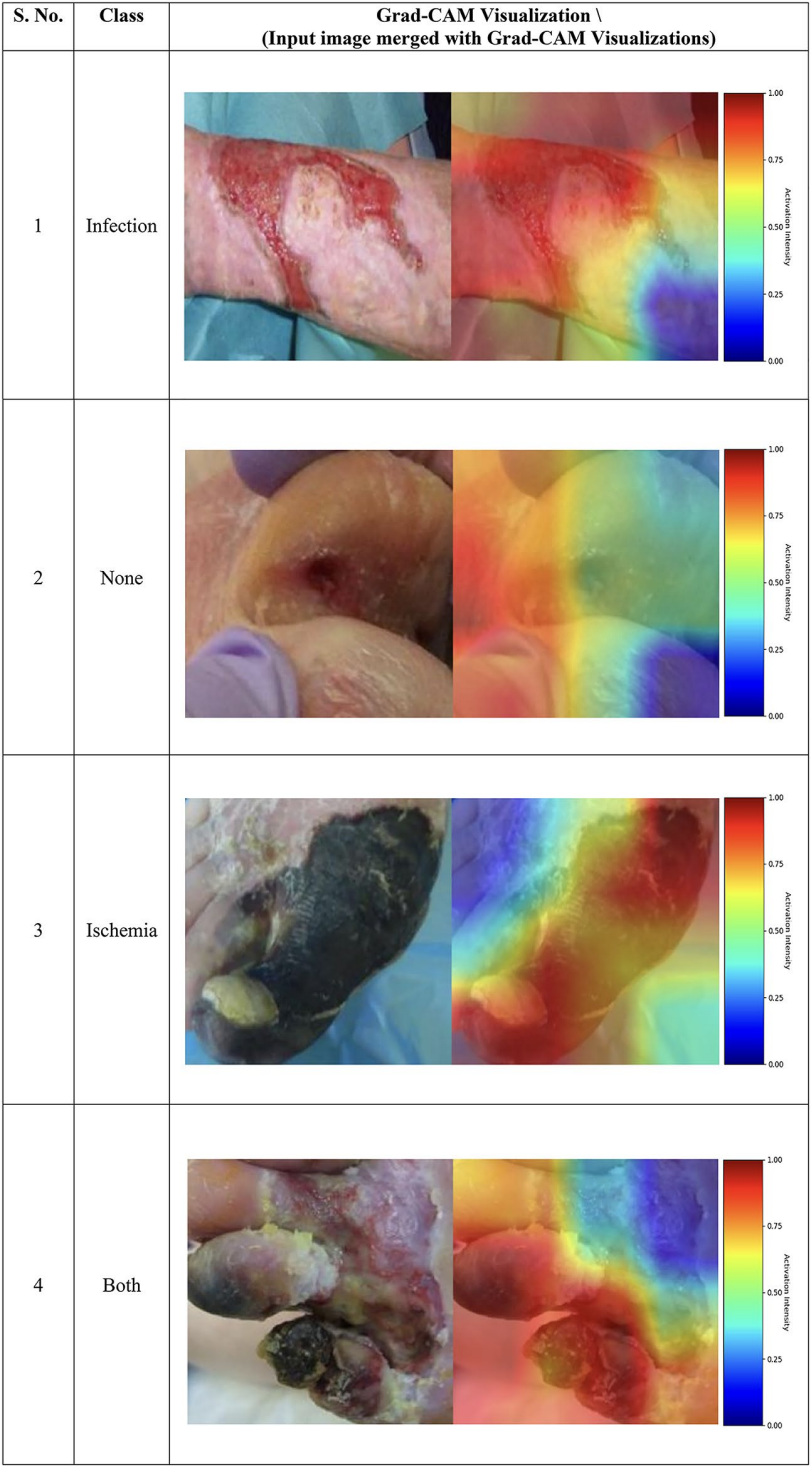
Visualization of the affected regions in the images of DFUC-2021 dataset across various classes using Grad-CAM.

The visualizations demonstrate how the proposed model adapts its attention across different DFU classes, focusing on relevant pathological regions. For example, in images classified as ‘Infection,’ Grad-CAM emphasizes regions showing active infections, while for ‘Ischemia,’ the attention shifts to areas showing reduced blood supply. In cases labeled as ‘Both,’ the heat maps exhibit activations in multiple regions corresponding to overlapping conditions. The above visualizations highlight how the proposed model focuses on different regions within the DFU images, offering valuable insights into its decision-making process. The Grad-CAM heat maps indicate the areas of the images that contribute most significantly to the model’s classification predictions. By examining these heat maps, medical practitioners can validate the model’s reasoning and ensure it is not relying on irrelevant artifacts or noise in the image. The colour bar is provided for reference to illustrate the intensity scale of the heat maps, where the color gradient ranges from blue to red. The color gradient bar is critical for interpreting the activation intensities, with blue indicating lower activation and red representing higher activation. Red areas signify regions of greatest importance to the model, offering a clear visual cue for the classification decision. The inclusion of the gradient bar ensures consistency and interpretability across all visualizations. This approach not only adds interpretability to the model but also promotes transparency, particularly in the context of medical image analysis for DFU classification.

### Performance comparison with state-of-the-art DL networks

In this section, we compare the performance of the proposed network with well-established DL models commonly used for image classification. We trained several state-of-the-art CNN models-AlexNet, ShuffleNet, ResNet, SqueezeNet, Xception, MobileNet, and DenseNet on the same dataset. Similarly, five transformers were trained-Pyramid Vision Transformer (PVT), Tokens-To-Token Transformer (T2T), Transformer-iN-Transformer (TNT), Vision Transformer (ViT), and Swin Transformer (SwinT). These pretrained architectures were fine-tuned to adapt to the DFUC-2021 dataset. The proposed network demonstrated significant improvements across all metrics compared to the pretrained DL models. Among the compared CNN architectures, DenseNet emerged as the top performing model with a macro F1-score of 79%, followed closely by ResNet with a macro F1-score of 78%. In contrast, MobileNet achieved a macro F1-score of only 56%. Among the compared transformer networks, SwinT achieved the highest macro F1-score with 68%, while PVT delivered subpar performance compared to the other transformer networks. SwinT was identified as the best performing transformer and was therefore used as the global feature extractor track in the proposed dual-track architecture. Detailed performance metrics for each model are provided in Table 11. The proposed network achieved a macro F1-score of 80%, outperforming all other models. These results highlight the ability of the proposed dual-path model in improving the precision of DFU classification.

**Table 11:** Performance comparison of the proposed network with state-of-the-art DL networks.

S. No.	Model	Number of parameters	Macro F1-Score (in %)
1	MobileNet	22,28,996	56
2	AlexNet	5,70,20,228	65
3	XceptionNet	2,08,15,148	69
4	SqueezeNet	7,37,476	77
5	ShuffleNet	12,56,679	78
6	ResNet	2,35,14,179	78
7	DenseNet	1,24,89,475	79
8	Pyramid vision transformer	2,39,74,916	40
9	Vision transformer	20,98,564	45
10	Tokens-To-Token Transformer	6,35,94,986	62
11	Transformer-in-transformer	2,34,60,388	66
12	Swin transformer	4,65,32,740	68
13	Proposed model	2,91,61,395	80

### Performance comparison with existing works

A comprehensive comparison of the proposed network with existing methodologies applied to the DFUC-2021 dataset is presented in Table 12. Previous studies have utilized various DL techniques, including transfer learning and ensemble learning, employing diverse architectures such as transformers and CNNs. The macro F1-score was used for comparison, as it was the only common metric reported across all studies, ensuring a fair and consistent evaluation. The proposed network emerged as the best-performing model, surpassing existing methods with better F1-score and accuracy metrics. This underscores the potential benefits of crafting DL models specifically tailored for DFU classification. By focusing on the intricacies of diabetic foot ulcer data, the proposed network can extract more relevant features and make more accurate classifications compared to existing architectures.

**Table 12:** Analysing the performance of the proposed network against existing works.

S. No	Source	Method	Macro F1-Score (in %)
1	Yap et al.^5^	Pretrained CNN	55
2	Qayyum et al.^20^	Transformer Network	56
3	Nakka et al.^14^	Pretrained CNN	59
4	Alavi et al.^17^	Custom CNN	59
5	Ahmed et al.^18^	Ensemble Learning	60
6	Wu et al.^16^	Pretrained CNN	60
7	Bloch et al.^19^	Ensemble Learning	61
8	Galdran et al.^22^	Custom CNN	62
9	Wu et al.^19^	Ensemble Learning	64
10	Proposed work	Swin Transformer with Custom CNN	80

### Performance evaluation across other datasets

This section evaluates the performance of the proposed model across various datasets to assess its generalizability. By testing on diverse datasets, the aim is to verify the model’s ability to adapt to different image distributions, ensuring robustness and applicability beyond the DFUC-2021 dataset. The results highlight the consistency and effectiveness of the proposed architecture when exposed to new data.

#### Evaluation on the DFU dataset

The proposed network was evaluated on the publicly available DFU dataset, which contains two classes: Healthy (543 images) and Ulcer (512 images)^37–39^. The Healthy class includes images of non-affected foot regions, ensuring a variety of skin tones and textures to account for diverse patient profiles. The Ulcer class comprises images of foot ulcers, capturing varying severities, shapes, and infection levels. This balanced dataset provides a robust platform for evaluating the model’s ability to classify both Healthy and Ulcer cases effectively. The proposed network demonstrated strong performance on this dataset, achieving an accuracy of 99%, precision of 98%, recall of 100%, and a macro F1-Score of 99%. These results highlight the generalizability and robustness of the proposed model when applied to external datasets.

#### Evaluation on the PAD-UFES-20 dataset

The proposed work was further evaluated on the PAD-UFES-20 dataset which consists of 2298 images from 1373 patients, representing six diagnostic categories: three skin cancers (Basal Cell Carcinoma (BCC), Squamous Cell Carcinoma (SCC), and Melanoma (MEL)) and three skin diseases (Actinic Keratosis (ACK), Nevus (NEV), and Seborrheic Keratosis (SEK))^40^. These images, collected during 2018 and 2019, include over 50 types of skin lesions, with the focus narrowed to the seven most common skin lesions for analysis. Bowens Disease (BOD) was grouped with SCC due to their clinical similarities, leaving six primary diagnostic categories. All skin cancer diagnoses in this dataset are biopsy-confirmed, ensuring high-quality labels. The proposed model achieved an accuracy of 90%, precision of 91%, recall of 89%, and macro F1-Score of 90% on the PAD-UFES-20 dataset. These results demonstrate the model’s capacity to generalize effectively to a more complex and diverse dataset with multiple diagnostic categories. The performance of the proposed network across different datasets is summarised in Table 13.

**Table 13:** Performance of proposed network across different datasets summarised.

S. No.	Dataset	Accuracy (in %)	Precision (in %)	Recall (in %)	Macro F1-Score (in %)
1	DFUC-2021	79	81	79	80
2	DFU	99	98	100	99
3	PAD-UFES-20	90	91	89	90

From the table above, it is evident that the proposed network performed consistently across different datasets, highlighting its robustness, adaptability, and effectiveness in handling diverse medical imaging scenarios.

## Conclusion

DFU pose a significant health threat, impacting the lives of millions of people worldwide and often leads to severe complications, including amputations. Early and accurate detection of DFU is crucial for timely intervention and effective management, yet this task remains challenging due to the complex and diverse characteristics of DFU. Conventional diagnosis methods can be time-consuming, subjective, and resource-intensive, placing a substantial burden on healthcare systems and medical professionals. The research presented in this work addresses these challenges by proposing an interpretable DL approach for the automated classification of DFU. The proposed architecture integrates a Swin Transformer track and a custom EMADN track, operating in parallel to extract global and local features simultaneously. The features are concatenated and processed through a Shuffle Attention mechanism to focus on informative regions, followed by GAP to reduce parameters.

The proposed network captures both high and low-level features, combining the contextual information and diverse feature learning capabilities of the Swin transformer. The integration of the CNN-based EMADN track with attention mechanisms enhance feature extraction and promotes model generalization. The inclusion of the SA module further refines the network’s ability to selectively focus on relevant information without additional computational overhead, leading to improved classification. The model was evaluated using the DFUC-2021 dataset and achieved a classification accuracy of 78.79% and a macro F1-score of 80%. Comparative analysis with various state-of-the-art DL models and existing works signifies the improved performance of the proposed network for DFU classification. Additionally, Grad-CAM was employed to produce visual interpretations to clarify the areas of focus for the model. The Grad-CAM analysis provides a transparent and explainable framework for adoption of the proposed model in real-world clinical applications.

## Data Availability

The data has been made available for DFUC 2021 participants. It is made available upon request by submitting a licence agreement to the dataset owner. Dataset can be requested through this link: https://helward.mmu.ac.uk/STAFF/M.Yap/dataset.php.
